# A Ghastly Experience

**Published:** 1887-12-31

**Authors:** 


### A Ghastly Experience.

I was dying. I knew I was dying. Yet my brain was as active as though I was in the full vigour of perfect health. At least I was quite conscious of all that was passing at the moment, though the past, even the events of the last few minutes, was a complete blank.

I was in bed in my own room at the hospital where I had held a post as ward sister. There was one person with me in the room, watching for my last breath. I could not see who my companion was; my eyes were fixed and glazed. The light had already gone from them.

The reason I knew I was in my own room was because I could distinguish the voices of the nurses and patients in the adjoining ward.

My breath each successive moment came in feebler and feebler gasps, with longer and longer pauses between. I thought, indeed, I had ceased to breathe, when another convulsive gasp caused my chest to heave and my jaw to relax once more.

A spoonful of some fluid was now put into my mouth. I could not taste or swallow, but I wondered, with a languid feeling of curiosity rather than with any personal interest in the matter, whether the fluid would choke me, and so hasten, by a moment or so, the last, last gasp.

I did not experience the slightest horror, or dread of death. I was in no pain, but felt only the sinking, exhausted sensation of fainting. But though I felt no horror of the grave or of the mysterious journey to the unknown land beyond, I acutely dreaded the coming ordeal of being prepared for burial.

I remembered vividly that some few nurses, hardened perhaps, by long and painful experience, did not always handle the dead with that gentleness with which they tended the living, and I felt much comforted at this moment to know that my conscience could not reproach me for ever having failed in due reverence to "senseless clay."

However, when the dreaded time came I was unconscious of it. How long this period of oblivion lasted I know not. When I next awoke I was sensible of being gently carried, and placed upon an operating table. The table was on a raised daïs in the middle of a large hall, lighted by a skylight immediately above the table. This hall was of considerable extent, a gallery ran round it, supported by white, gleaming columns, resembling marble. The hall in no way resembled the operating theatre of any hospital I had seen. Having been trained as a nurse in one of the largest hospitals in London, I was quite familiar with the ordinary appearance of such chambers of horror. My impression was that I was in the lecture hall of a very large colonial hospital. The hall was crowded with doctors and students, of whom a small proportion were women. All who were near enough crowded round to look at me. They were talking, joking, and jostling each other, while one or two, whose voices sounded familiar to me, remarked: "Poor thing! How sudden! I wonder what caused it!" or words to that effect.

Presently an increased commotion was heard proceeding from an adjoining corridor. At the sound, the chattering voices in the hall were hushed; every eye was turned in the direction from whence the approaching sounds proceeded. A swinging glass door was pushed roughly back, and through the opening there advanced a tall, portly, fair woman of middle age, followed by several nurses, who were apparently endeavouring to prevail upon her to retire. The woman, from her dress and appearance, might have been the matron. She was talking loudly, in a high-pitched shrill voice, and was evidently greatly excited. Advancing rapidly through the crowd, which made way at her approach, she said first to one, then to another, without waiting for a reply, "Where is Dr. R—— ? Tell him I want him."

"I am here, madam," answered the gentleman in question. "What can I do for you?"

"Do for me!" repeated the woman wildly. "Why retract here, in public, the frightful accusation you have brought against me. *That* is what you must do."

"But suppose I refuse to retract one word. Suppose, on the contrary, I accuse you here, publicly, of causing the death of the unfortunate lady whose body lies here before us. What then, madam?"

"In that case I should say your accusation was a wicked fabrication, without a shadow of foundation."

"Then so be it, madam; and I should advise you to set about collecting proofs of your innocence without delay."

"Thank you for your advice; I shall act upon it immediately," answered the lady; but her last words were almost inaudible. The excitement which had hitherto sustained her was dying out. Her flushed cheek paled, her mouth quivered, while she glanced nervously around and became painfully aware of the pitiless, stern expression of the faces surrounding her.

Then she turned and retreated—some might say fled—her exit being even more swift and sudden than her entrance.

Once more they raised and carried me. I wondered if I was being conveyed to the room in the mortuary where post-mortems were held. Even this reflection did not terrify me. On the contrary, I felt that it would be a grim sort of satisfaction to know the cause of my death.

And now comes a very strange incident. In carrying my body, enveloped as it was in a sheet, a pin which fastened it together ran into me. Marvellous to relate, this pin piercing my dead flesh caused me exquisite pain. It not only pained, but surprised me exceedingly. "Was it possible," I thought, "that the nerves of sensation do not die at once. But indeed, why should they when my brain is still alive and active?" How intensely I longed at that moment to come to life again, just for a few minutes, that I might tell the doctors what I experienced. What light might not such information throw upon science? But the pain that pin was causing once more absorbed me entirely. With a supreme effort of will I endeavoured to move even a finger which was close to the offending pin in order to remove it. But no. Not a muscle could I move. Fortunately, however, relief came in an unexpected manner. In shifting his hand to get a firmer grasp of my shoulder the man who was assisting to carry me dragged upon the sheet, and so displaced the instrument of torture. The instant it was withdrawn the pain entirely ceased. There was no tingling sensation whatever remaining. The pain went as suddenly as it came.

Again my brain busied itself with reflections as to whether it were not a merciful dispensation of providence that human beings were almost universally inspired with an instinctive dread of roughly handling the dead. "Of course they cannot know that we feel after we are dead, so why should they be tender with us?" I reflected peacefully. Again came the thought of a possible post-mortem. Still the idea did not trouble me, though I had been so recently and painfully convinced that the nerves of sensation were as sensitive as when I lived and breathed. "Doubtless there is some wise provision against our suffering under such circumstances," I mentally decided.

I now felt that I was being carefully deposited upon a kind of bier, or stretcher—which, by-the-by, ought to have been used in moving me hither; this was on low trestles not more than a foot in height. And now came another interval of complete unconsciousness, from which condition I suddenly re-awoke, and became sensible of the rustling and confused sounds of the approach of a large number of people. They came nearer and nearer, until I was once more the centre of a crowd of spectators. It seemed strange to me that though my eyes were closed, though I could not by any effort of will have lifted an eyelid, I could see and distinguish those around me. Most of them were students who walked the wards of our hospitals; but the central figure around whom they clustered, and who evidently was the surgeon who was to conduct the autopsy, was a stranger to me. Though I saw him only this once, I could tell every detail of his appearance now if it were necessary; and I watched with rapt attention while he selected from his case of instruments the knife with which to make the first incision on my helpless body.

There was a good deal of talk and jesting going on, and I heard one student make a wager with another as to whether my death was due to poison or to some natural cause. Their lightness wounded me; it seemed so strangely callous in the presence of the awful mystery of death. Suddenly across the chatter came a voice, clear and distinct, that uttered the familiar Gospel words, "She is not dead, but sleepeth." The tone was very quiet, and lower than that of ordinary conversation; only its distinctness made it more than a whisper; and first I wondered how, coming as it seemed to do from a corner of the room, I could hear it so well; then I asked myself if those around me heard it, or if it only spoke to my soul's ear.

But apparently it was a mortal voice, for a sudden awed silence fell on the assembly, and all eyes were turned to the corner whence it came. I, too, strained my vision to see, but I could distinguish no one, even when, in response to a scornful request on the part of the surgeon, that "the gentleman who held that strange opinion would step forward and explain his reasons for it," I knew—I saw—the spectators make way for him to pass into the middle of the room. I saw nothing, as I say, yet I was conscious, with some knowledge beyond sight, that he who had spoken came and stood opposite the surgeon at the other side of my bier, and that his eyes were fixed upon my face. I cannot describe that look. It pierced through the cold enveloping flesh to the still active brain, and sent a thrill from the keen sentient nerves through every muscle of my body. I still believed that I was dead; but a warmth different from that of mere living blood permeated my veins, and I began to dread with an agonising horror that investigation by the surgeon's knife to which I had been a few minutes before coldly acquiescent. Dead as I was, I knew that I could move, breathe, feel at this new-comer's bidding.

He spoke again; this time to the operator. "There is no need for me to explain my opinion," he said; "the event will prove it to be right."

"That's a very unscientific way of speaking," answered the surgeon, "and I have too much to do to-day to postpone the examination till 'the event' proves you to be either right or egregiously wrong."

"As you like," replied the other—my master—quietly; but even while he uttered the words I felt a stronger effluence of life and strength pass from his eyes into mine. He said no more, however, and the operator, turning to the students, remarked, with a smile, "If you like, gentlemen, I will preface my more serious investigations with an 'exploratory puncture,' just to satisfy this very self-willed person that his opinion is wrong."

A murmur of applause and laughter followed this observation, and the operator bent forward and plunged his lancet into my arm. I felt no pain, though a stream of warm, red blood spurted living from the wound; but the incision tore away the last remnant of the torpor that had held me. Dead or living, I could move again—drawn on, supported by my master's gaze.

I raised myself without assistance, sat up, opened my eyes, and saw before me a tall, thin man with a very intelligent face, lighted up with the brightest and most piercing eyes I ever beheld.

Under the mesmeric influence of his will I arose and walked forward, a large crowd of spectators separating eagerly to allow me to pass.

When I had traversed about half of the vestibule I suddenly came face to face with the supposed matron, who had been accused of poisoning me. At the sight of me advancing towards her she threw up her arms, her face became white and rigid as my own, and she would have fallen had not those near her supported her. At the will of my master I advanced close to her in spite of her convulsive struggles to avoid me, and when quite near her I, too, threw up my arms, and uttered three piercing shrieks, being fully conscious at the moment that I was re-enacting the scene which immediately preceded the stupor which ended in my death.

In the act of uttering the last shriek I fell forward, and simultaneously awoke to find I had been—only dreaming.

